# CIN grades possessing different HPV RNA location patterns and RNAscope is helpful tool for distinguishing squamous intraepithelial lesions in difficult cervical cases

**DOI:** 10.1186/s13000-023-01308-w

**Published:** 2023-02-16

**Authors:** Ruichao Chen, Renchao Zhang, Minfen Zhang, Shaoyan Liu, Mingyu Xie, Zhongfeng Yang, Quan Shi, Hui Chen, Hanzhen Xiong, Na Wang, Qingping Jiang

**Affiliations:** 1 Department of Pathology, the Third Affiliated Hospital, Guangzhou Medical University, Guangzhou, China; 2 Guangdong Provincial Key Laboratory of Major Obstetric Diseases, Guangzhou, China; 3 Department of Pathology, University of Chinese Academy of Sciences-Shenzhen Hospital (Guang Ming), Shenzhen, China; 4 Department of Pathology, The First Hospital of Changsha, Hunan, China; 5 Department of Pathology, Guangzhou Women And Children’s Medical Center, Guangzhou, China

**Keywords:** Cervical intraepithelial neoplasia, Human papilloma virus, RNAscope

## Abstract

**Background and objectives:**

The precise grading and characterization of cervical intraepithelial neoplasia (CIN) has been the focus of pathologists for a long time. This study aimed to explore known strategies for the grading of CINs.

**Methods:**

After routine H&E review, 85 lesions graded CIN 1, 2, or 3 were investigated primarily by HPV RNAscope to detect HR-HPV and LR-HPV, in combination with an HPV-DNA test and P16/Ki67 immunohistochemistry (IHC). Then, the 85 cases were divided into a control group (49 cases) and a test group (36 cases). The former consisted of cases with consistency between morphology, HPV DNA detection and P16/Ki67 IHC. We used them to evaluate HPV RNA distribution patterns in CINs of different grades. The latter were ambiguous cases in which pathologists could not confirm the diagnosis because of inconsistencies between morphology, HPV DNA detection and P16/Ki67 IHC. We reassessed them by comparison to the pattern in the control group.

**Results:**

The expression patterns of HPV mRNA signals were different in different CIN lesions. LSIL/CIN1 lesions were mostly expressed in superficial epithelium with diffuse clustered nuclear or cytoplasmic staining; HSIL/CIN2 were characterised by nuclear/cytoplasmic punctate or diffuse cluster nuclear staining in the mid-surface layer, and scattered nuclear/cytoplasmic punctate staining in basal and parabasal cells; whereas HSIL/CIN3 showed full-thickness nucleus/cytoplasmic scattered staining with a punctate pattern. According to the staining pattern, we corrected the diagnosis of 22 cases (22/36, 61.1%).

**Conclusion:**

Because of its distinct location pattern, HPV RNAscope has obvious advantages over the HPV-DNA test, and combined with P16/Ki67 IHC, it can help pathologists correctly grade CIN. In addition, it can effectively discriminate true CIN from normal or CIN mimic lesions, such as immature squamous metaplasia, atrophy, and inflammatory/reactive changes. Therefore, HPV RNAscope is a valuable auxiliary diagnostic test to avoid the overtreatment and undertreatment of CIN lesions.

**Supplementary Information:**

The online version contains supplementary material available at 10.1186/s13000-023-01308-w.

## Introduction

Uterine cervical carcinoma (UCC) is one of the three major malignant tumours in gynaecology and seriously threatens the health of women. Approximately 14,480 women in the United States are diagnosed with cervical carcinoma, and 4,290 die from the disease [1]. In China, the morbidity of cervical carcinoma has become the second highest among female malignancies, just behind breast carcinoma [2].

For patients to go from HPV infection to UCC involves a lengthy process that provides time for early detection and intervention [3, 4]. Cervical carcinoma prevention programs in China have mainly relied on a three-stage intervention: 1) Pap tests/cervical cytology for screening; 2) colposcopic evaluation and cervical biopsy; and 3) loop electrosurgical excision procedure (LEEP) or excisional treatment of cervical in women diagnosed with cervical intraepithelial neoplasia(CIN) or early-stage UCC [5]. The clinical treatment strategies for CINs are based on pathological diagnosis. Based on cervical histopathological diagnoses, CIN can be divided into low-grade squamous intraepithelial lesions (LSIL/CIN1) and high-grade squamous intraepithelial lesions (HSIL/CIN2, CIN3). Previously, the classification of CIN was mainly based on a subjective measure of the degree of epithelial involvement from the basement to the surface. CIN1 is characterized by the involvement of the lower third of the epithelium, which shows proliferation of basal/parabasal-like cells; CIN2 involves between one-third and two-thirds of the epithelium, and CIN3 involves more than two-thirds [6]. However, in recent years, increasing evidence has confirmed that LSIL and HISL are two different and noncontinuous biological processes. The latter is a precancerous lesion in which the tumour cells proliferate clonally, while the former is a HPV infection that still possesses a normal stratified epithelial structure. Distinguishing between HSIL and LSIL is essential for clinical management. Therefore, a lesion cannot simply be classified into a certain grade by the percentage of epithelium involved [7]. The World Health Organization (WHO) also recommends that the two-tiered LSIL/HSIL system be the preferred terminology in both tissue and cytology specimens from the cervix, because it has improved reproducibility and biological relevance compared to the previous three-tiered CIN 1/2/3 system [8]. However, regardless of the category, it has always been a dilemma to accurately distinguish CIN1 and CIN2 [9, 10]. Moreover, distinguishing CIN2 from CIN3 is also important because CIN2 has a greater regressive potential and less progression than CIN3. In 2018, Tainio et al. found there were up to 50% CIN2 cases spontaneously regressed after 24-months’ follow-up [11]. In 2021, 55% cases of CIN2 were found regression, while 28% cases of CIN3 regression [12]. In young women (age 21 to 25), when 40% of CIN2 and CIN3 regressed in 6 months, up to 71% of CIN2 regressed. Therefore, in management of young women with CIN2, they could be considered follow-up for up to 24 months and treatment only if the HSIL persists. CIN2 provides a necessary margin of safety for the patient, and the gynaecologist can fully provide treatment measures according to the patient’s condition.

At present, the pathogenesis of cervical carcinoma is relatively clear, and a large number of UCC and CIN cases are related to a persistent infection of high-risk human papilloma virus (HR-HPV). In particular, HPV16 and HPV18 are the most common types, detected in approximately 50 and 20% of cervical squamous cell carcinomas, respectively [13]. The HPV family can bind to P53 by encoding E6 and E7 proteins, leading to the degradation and cell transformation of P53 and promoting the occurrence of carcinoma. In addition, HPV DNA can be integrated into the host cell genome, leading to the inactivation of the tumour suppressor gene product Rb, thus increasing the E2F-1 level and overexpressing P16 [14, 15]. In pathological routine work, HPV-DNA tests can detect whether patients have an HPV infection. However, the tests can only be performed on liquid cytology preparation and HPV-DNA cannot be located on biopsied tissues, which makes our diagnosis difficult.

In formalin-fixed paraffin-embedded (FFPE) tissue samples, the biomarkers P16 and Ki-67 are common and helpful tools widely used to guide CIN grading by pathologists [16–18]. In general, P16 staining is negative or patchy-like positive in cervical normal squamous epithelium and LSILs, and block-positive in almost all HISLs and some LSILs. Ki67 gradually increases from CIN1 to CIN3. However, chronic cervicitis, pregnancy or benign squamous epithelium with reactivity may mimic SIL with high Ki67 expression. And conversely, in rare occasions, P16 is negative in HSIL cases [19, 20]. In addition, because atrophy and/or immature metaplasia share some morphological features with HSIL, and P16 may block positive expression in these cases, they may be overdiagnosed as HSIL [21, 22]. These conditions are not uncommon in our routine diagnosis. This situation has prompted us to search for more effective methods to verify the degree of cervical lesions and avoid over- or underdiagnosis.

As stated earlier, persistent HR HPV infection plays a key role in the development of CIN and UCC. In recent years, a new detection method named HPV E6/E7 mRNA In Situ Hybridization (RNAscope) has appeared. In this study, we used HR-HPV and LR-HPV RNAscope to analyse 85 cervical lesions, compared them with the results of HPV DNA cervical cytology tests and P16/Ki67 immunohistochemistry, and performed the pathological diagnosis.

We divided 85 CIN lesions into two groups: the control group (H&E morphology is consistent with HPV-DNA and P16/Ki67 immunotype) and the test group (one or two results in total did not match the SIL in morphology, HPV-DNA and P16/Ki67 immunotype). Using these combined assays, we not only found that HPV RNAscope has different expression patterns in different grades of CIN, but more importantly, HPV RNAscope is an effective auxiliary diagnostic tool for difficult CIN grading cases. HPV RNAscope can provide a more accurate diagnosis of CIN lesions, which can help detect HPV types that have not been included in our routine detection, and can greatly reduce the overdiagnosis or underdiagnosis of cervical lesions.

## Materials and methods

### Specimen collection

This was a retrospective observational cohort study. We screened 85 cases of CIN lesions at the Third Affiliated Hospital of Guangzhou Medical University: CIN1 (*n* = 29), CIN2 (*n* = 31), and CIN3 (*n* = 25) from 2019 to 2022. The age of the patients ranged from 20 to 64 years old, and they had no other diseases in female reproductive tract. The clinical data and pathological characteristics of 85 CIN cases are shown in Table 1. All cases contained TCT, HPV-DNA genotyping, P16/Ki-67 immunohistochemical staining, and cervical biopsy pathology results. All cases were reviewed and regraded by two specific gynaecological pathologists. The study was approved by the ethics committee of Third Affiliated Hospital of Guangzhou Medical University.

**Table 1:** The clinical data and pathological characteristics of 85 CIN cases

Parameter	Number	Percentage
Ages	20–64	40.4 ± 11.1
Pre-menopause	77	90.60%
Post-menopause	8	9.40%
HR-HPV positive genotype	72	84.70%
HPV16	9	10.60%
HPV18	8	9.40%
^a^Other 12 HR-HPV types	45	52.90%
HPV16 + 18	2	2.40%
HPV16 + Other 12 types	6	7.10%
HPV16 + 18 + Other 12 types	2	2.40%
HR-HPV negative genotype	13	15.30%
P16		
Positive	48	56.50%
Negative	37	43.50%
Ki67		
High expression	45	52.90%
Low expression	40	47.10%
CIN		
CIN1	29	34.10%
CIN2	31	36.50%
CIN3	25	29.40%
Grouping		
Control	49	57.60%
Test	36	42.40%

^a^The other 12 HR-HPV types mean HPV-31, 33, 35, 39, 45, 51, 52, 56, 58, 59, 66 and 68

### HPV testing and cytology

The HPV-DNA test was performed following the manufacturer’s instructions using a Cobas®4800 system liquid cytology preparation kit (Roche Molecular Systems Inc, Basel, Switzerland), which can examine 14 high-risk HPVs at one time, including types 16, 18, 31, 33, 35, 39, 45, 51, 52, 56, 58, 59, 66 and 68. Pap smear samples were collected in preserved fixative solution, and preserved slides were prepared, stained, and processed with an automated stainer. The TCT slide results were confirmed by two pathologists who specialize in cytology.

### Immunohistochemistry (IHC)

IHC for P16 (clone: ZM-0205, ZSGB-BIO, China) and Ki67 (clone: MIB1, TALENT-BIO, China) was performed according to the manufacturer’s instructions. After deparaffinization and dehydration, antigen retrieval was performed by boiling the slides with ethylene diamine tetraacetic acid (EDTA) antigen retrieval solution in a pressure cooker for 15 min. Then, the sections were incubated with the antibody after blocking endogenous peroxidase with H_2_O_2_ followed by incubation with the secondary antibody. The reaction was detected by diaminobenzidine (DAB), and the slides were redyed with haematoxylin–eosin (H&E) staining. Meanwhile, positive and negative control slides were designed to ensure the effectiveness of the staining procedure. Finally, the slices were put into xylene solution for transparency, sealed with neutral gum, and observed under a bright field microscope.

According to the Lower Anogenital Squamous Terminology (LAST) [23], P16 positivity is defined as continuous staining called “block positivity” in the nucleus and/or cytoplasm, which extends from the basal layer through at least one-third of the epithelium. Ki67 positivity is defined according to CIN grade. Ki67- is defined as staining of nuclei in the basal or parabasal layer; CIN1/Ki67 + is defined as continuous nuclei staining extending from the basal layer to the lower third of the epithelium; CIN2/Ki67 + is defined as positive nuclei staining predominantly found in the lower two-thirds of the epithelium. CIN3/Ki67 + defined as positive nuclei staining in more than two-thirds of the epithelium. All of the slides were reviewed by two trained pathologists.

### RNAscope detection

RNAscope® 2.5 HD Detection Reagent was purchased from Advanced Cell Diagnostics (ACD, Newark, CA). Experimental procedures were strictly implemented in accordance with the instructions provided by ACD. Briefly, the samples were fully fixed (10% formalin at room temperature for 16–24 h), sliced, deparaffinized, and dehydrated in sequence. Sections were then treated serially with Pre-Treatment 1–3 solution and rinsed with distilled water after each Pre-Treatment step. The sections without a cover slip were then hybridized in HPV hybridization solution at 40 °C for 30 min in a HybEZ Oven (ACD, Newark, CA). The hybridized probe signal was amplified through the serial application of Amp 1–6; wash buffer steps were performed after each step. The signal intensity was demonstrated by the application of diaminobenzidine (DAB) for 10 min at room temperature. Finally, sections were counterstained with haematoxylin, dehydrated through graded ethanol and xylene and then mounted for microscope observation.

The HPV RNA signal appeared brown in the nucleus or cytoplasm on the sections, and normal epithelium was used as an internal negative control. Staining data were recorded as positive patterns based on factors such as signal intensity, the range of staining, the thickness of epithelial staining, and cytoplasm/nucleus staining. All of the slides were reviewed by two trained pathologists.

### Statistical analyses

Statistical analyses were performed using SPSS software (17.0). The expressed ratios and percentages were used to describe the measures in the study. The comparison of the enumeration data between the control group and test group was performed by the row × list chi-square test, and a *P* value < 0.05 was considered statistically significant.

## Results

### The design of the control group and test group

First, all CIN lesions were divided into the CIN1 group (*n* = 29), CIN2 group (*n* = 31) and CIN3 group (*n* = 25) according to the original pathological diagnosis. Then, according to the differences in P16/Ki-67 IHC and HPV-DNA results, the CIN lesions in the 3 groups were further separated into 2 groups: the control group and the test group. The control group included cases in which HPV DNA and P16/Ki67 phenotype matched the H&E morphology, whereas those that were not matched were included in the test group. Based on the different inconsistent combination of HPV-DNA and P16/Ki67 phenotypes, the test group was further divided into three groups: A (P16-, Ki67 + and HPV +), B (P16 + , Ki67- and HPV +), and C (P16 + , Ki67 + and HPV-). The number of cases in each group is shown in Table 2. The control group was used to define the HPV RNA location pattern in different CIN grades, and then according to those patterns the final diagnosis for the test group was determined.

**Table 2:** The number of cases in each group

	Control	Test-A	Test-B	Test-C	Total
CIN1	16	4	2	7	29
CIN2	15	6	5	5	31
CIN3	18	3	1	3	25
Total	49	13	8	15	85

Control group: P16 + , Ki-67 + , HPV +

Test-A group: P16-, Ki-67 + , HPV +

Test-B group: P16 + , Ki-67- or < 10%, HPV +

Test-C group: P16 + , Ki-67 + , HPV- or P16-, Ki-67-, HPV +

### The location characteristics of HPV RNA is distinctive in different CIN grades in the control group

**Fig. 1:**
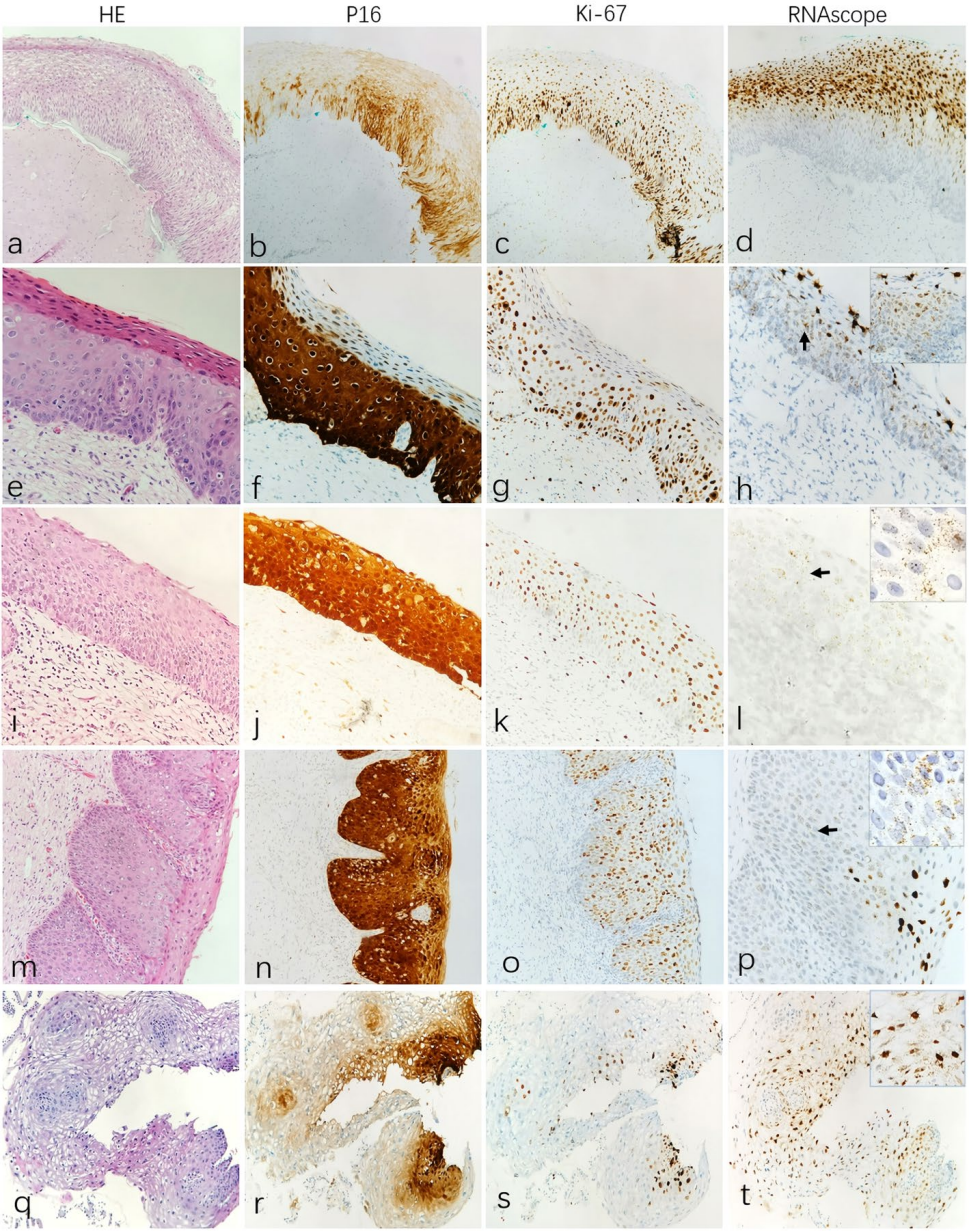
The Location Character of HPV RNA in different CIN grades in the control group. Row1 (**a**-**d**): HR-HPV RNA in CIN1 lesion (P16 + and Ki67 +) show diffuse clustered expression pattern in nuclei/cytoplasmic, staining areas is mainly located on the surface epithelium (**d**). Row2 (**e**–**h**): HR-HPV RNA in CIN2 lesion (P16 + and Ki67 +) show nucleus/cytoplasmic dot positivity in the basal and parabasal layer, and combined with diffuse cluster nuclei positivity on the surface epithelium (**h**, arrow). Row3 (**i**-**l**) and Row4 (**m**-**p**): HR-HPV RNA in CIN3 lesion (P16 + and Ki67 +) show dotted full-thickness nucleus/cytoplasmic scattered staining(l, arrow) or combined with a clustered nuclei staining on the superficial cells (bottom right of image **p**). Row5 (**q**-**t**): The expression pattern of LR-HPV RNA in CIN1 lesion (P16 + and Ki67 +) was like HR-HPV, showing diffuse clustered expression pattern in nuclei/cytoplasmic, staining areas is mainly located on the surface epithelium (**t**, arrow)

There were 49 CINs in the control group, including 16 cases of CIN1, 15 cases of CIN2, and 18 cases of CIN3. HR-HPV RNAscope detection was performed in this group, and all 49 cases were positive (100%). In 16 cases of CIN1 lesions, a diffuse clustered expression pattern in nuclei or cytoplasm was observed; staining areas were mainly located on the upper 1/3–1/2 of the epithelium (Fig. 1, d), and 3 cases showed weak to strong spot staining on the basal or parabasal layer at the same time. Among the 15 cases of CIN2 lesions, 14 cases showed nucleus/cytoplasmic dot positivity in the basal and parabasal layer, and 13 cases were combined with diffuse cluster nuclei positivity on the upper 1/3–1/2 of the epithelium (Fig. 1, h). The remaining 1 case only showed dotted nuclei/cytoplasmic positivity in the basal and parabasal layers but no cluster nuclei on the upper 1/3–1/2 of the epithelium. Similarly, an HR-HPV mRNA positive signal could be detected in all CIN3 cases in the control group, of which 16 cases had full-thickness nucleus/cytoplasmic scattered staining in the dotted region (Fig. 1, I), and 1 case was combined with clustered nuclei staining on the superficial cells (Fig. 1, p). The other 2 cases only showed scattered nuclear/cytoplasmic spots in the basal and parabasal layers or CIN3-involving endocervical glands. The distribution characteristics of HR-HPV RNAscope expression patterns for different CIN lesions in the control group are shown in Table 3.

**Table 3:** Distribution characteristics of HR-HPVRNAscope expression patterns for different CIN lesions in the control group

RNAscope +	CIN1(*n* = 16)	CIN2(*n* = 15)	CIN3(*n* = 18)	χ^2^	*P* value^a^
BME +	0/16(0)	1/15(6.7%)	2/18(11.1%)	1.831	0.4
FTE +	3/16(18.8%)	13/15(86.7%)	16/18(88.9%)	22.745	< 0.001
SLE +	16/16(100%)	14/15(93.3%)	1/18(5.6%)	40.92	< 0.001

*BME* Basal to mid-epithelium, *FTE* Full thickness epithelium, *SLE* Superficial layer epithelium

^a^chi-square test

In terms of LR-HPV RNAscope detection, only 2 CIN1 cases were also found to have an LR-HPV infection in addition to HR-HPV in the total 49 cases. The expression pattern was similar to that of HR-HPV, which was diffuse and clustered in nuclei/cytoplasm, and the staining areas were mainly located on the upper 1/3–1/2 of the epithelium (Fig. 1, t). No LR-positive cases were found in CIN2 or CIN3.

Statistically, it could be seen that the dotted expression on the full thickness epithelium (FTE) staining pattern in CIN2 (86.7%) and CIN3 (88.9%) was significantly higher than in CIN1 (18.8%). In addition, the frequency of clustered expression on the superficial layer epithelium (SLE) staining of CIN1 (100%) was significantly higher than that of CIN3 (5.6%). In conclusion, the results of the control group showed that there were different expression patterns of HPV mRNA in different CIN lesions. The main staining features of LSIL/CIN1 were diffuse clustered nuclear and cytoplasmic staining of the superficial epithelium. HSIL/CIN2 showed nuclear/cytoplasmic punctate or diffuse cluster nuclear staining in the mid-surface layer and scattered nuclear/cytoplasmic punctate staining in basal and parabasal cells. HSIL/CIN3 showed full-thickness nucleus/cytoplasmic scattered punctate staining.

### Analysis of HPV RNA expression in the tested group and discriminate between true CIN and normal or CIN mimic lesions

HR-HPV and LR-HPV RNAscope detection was performed on 36 cases in the test group, of which 16 cases were HR-HPV positive and 1 case was LR-HPV positive, with a positive rate of 47.2%. The number of CIN1-CIN3 cases and RNAscope-positive cases in the test group are summarized in Table 4.

**Table 4:** The number of CIN1-CIN3 cases and RNAscope positive cases in the test group

	RNAscope + cases/Total			Total
	CIN1	CIN2	CIN3	
Test-A(*n* = 13)	2/4	1/6	3^a^/3	6/13
Test-B(*n* = 8)	1/2	2/5	1/1	4/8
Test-C(*n* = 15)	2^b^/7	1/5	3/3	6/15
Total(*n* = 35)	5/13	4/16	6/6	15/35

Test-A group: P16-, Ki-67 + , HPV +

Test-B group: P16 + , Ki-67- or < 10%, HPV +

Test-C group: P16 + , Ki-67 + , HPV- or P16-, Ki-67-, HPV +

^a^P16 mottled positive at CIN3 was classified as group Test-A

^b^This group included 1 case of LR-HPV-HPV

#### Test-A group

**Fig. 2:**
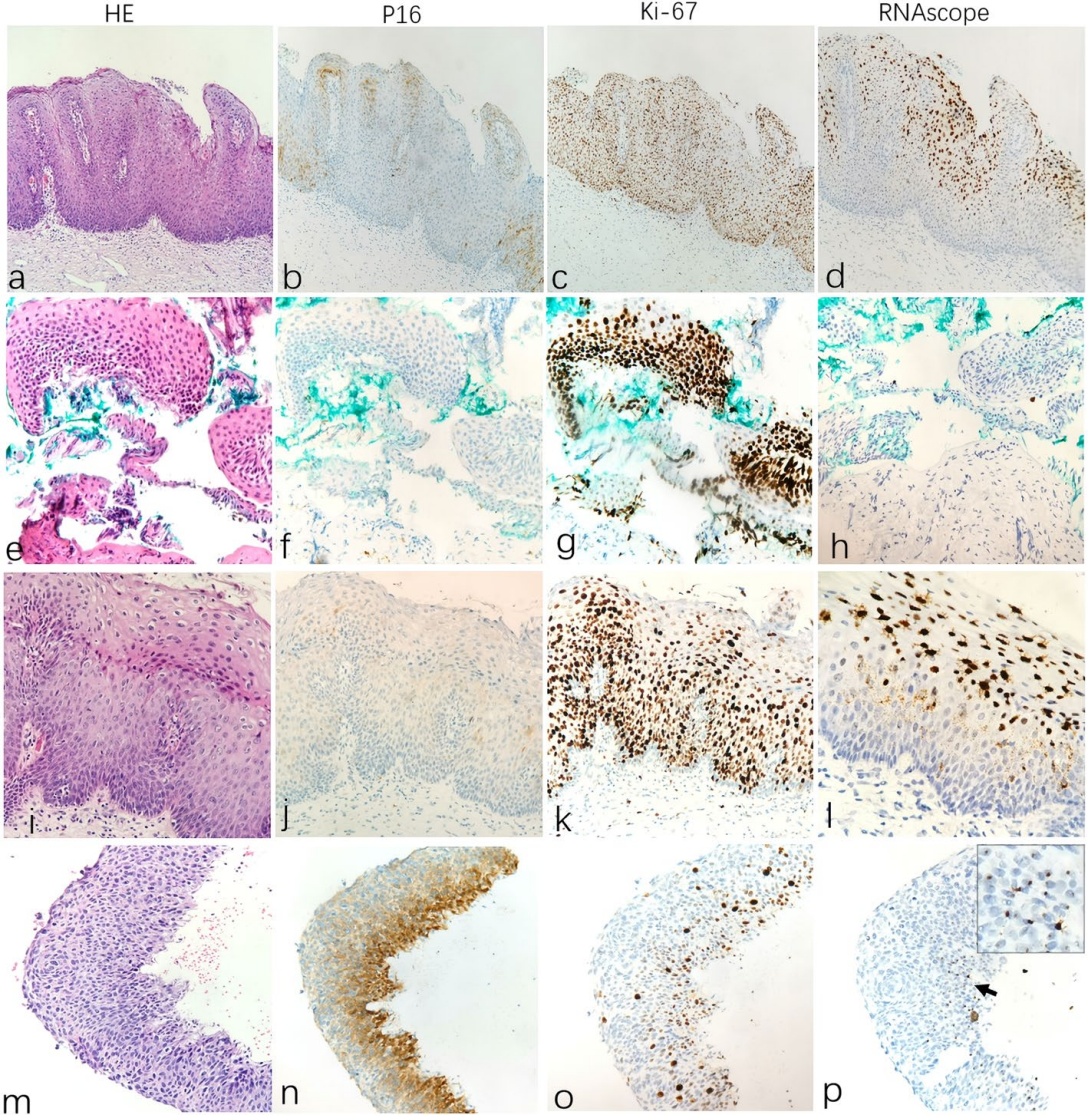
The H&E morphology, P16/Ki67 phenotype and RNAscope expression patterns in Test-A group. Row1 (**a**-**d**): In P16- CIN1 case, RNAscope show diffuse clustered expression pattern in nuclei/cytoplasmic, staining areas is mainly located on the surface epithelium (**d**). Row2 (**e**–**h**): P16- CIN1 cases but RNAscope is negative (**h**). Row3 (**i**-**l**): In P16- CIN2 case, RNAscope show diffuse cluster positivity and cytoplasmic punctate positivity in superficial cells and scattered punctate positivity in basal and parabasal nuclei/cytoplasmic (**l**). Row4 (**m**-**p**): In P16- CIN3 case, RNAscope show dotted full-thickness nucleus/cytoplasmic scattered staining (**p**, arrow)

There were 13 CIN samples in the test-A group, in all of which the auxiliary examination showed P16-, Ki67 + and HPV + staining. The H&E morphology, P16/Ki67 phenotype and RNAscope expression patterns are shown in Fig. 2. Among the original 4 CIN1 cases, 2 were positive for HR-HPV RNAscope and consistent with the CIN1 RNAscope staining characteristics of the control group (SLE staining pattern, Fig. 2, d), which confirmed the original CIN1 diagnosis. However, the other 2 cases were negative for HR-HPV mRNA and LR-HPV, and CIN1 was finally corrected to inflammation after pathologists reviewed the H&E slides repeatedly and combined them with the relevant examination (Fig. 2, h). For the original 6 CIN2 samples, only 1 was HR-HPV positive, which was characterized by RNAscope staining of CIN2 in the control group, namely, diffuse cluster positivity and cytoplasmic punctate positivity in superficial cells and scattered punctate positivity in basal and parabasal nuclei/cytoplasm (Fig. 2, l), which was consistent with the original CIN2 diagnosis. However, no RNAscope positivity was found in the other five cases, suggesting that there was no HPV E6/E7 mRNA transcription, and all of them were corrected to be inflammation or reparative changes. Three cases of CIN3 lesions were all HR-HPV positive and consistent with the CIN3 RNAscope staining characteristics of the control group, including 2 cases with scattered punctate signals in full-thickness nuclei/cytoplasm (Fig. 2, p) and the other case with scattered punctate signals in the CIN3-involving gland area.

#### Test-B group

**Fig. 3:**
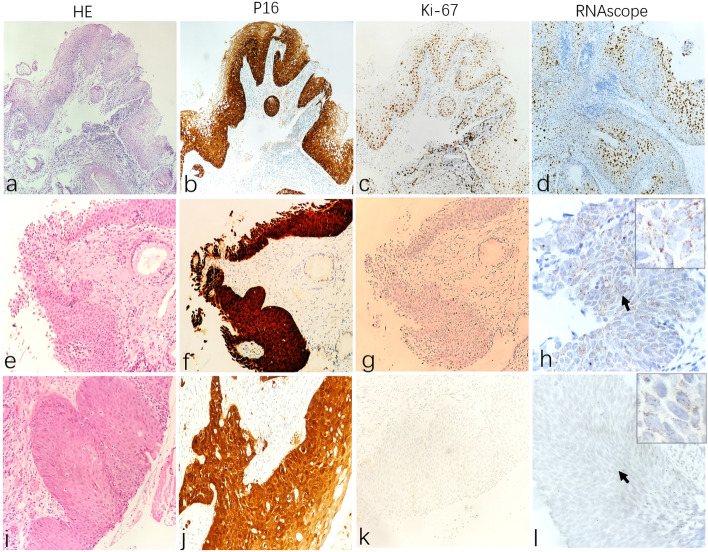
The H&E morphology, P16/Ki67 phenotype and RNAscope expression patterns in Test-B group. Row1 (**a**-**d**): In CIN1 case with low expression of Ki67, RNAscope show diffuse clustered expression pattern in nuclei/cytoplasmic, and the staining areas is mainly located on the surface epithelium (**d**). Row2 (**e**–**h**): In case originally diagnosed as CIN2 with low expression of Ki67, RNAscope show dotted full-thickness nucleus/cytoplasmic scattered staining (**h**, arrow). Row3 (**i**-**l**): In CIN3 cases with low expression of Ki67, RNAscope show dotted full-thickness nucleus/cytoplasmic scattered staining (**l**, arrow)

The phenotype of 8 original CIN samples in group Test-B was P16 + , Ki67- and HPV + . The H&E morphology, P16/Ki67 phenotype and HPV-RNAscope expression patterns are shown in Fig. 3. In 2 cases of CIN1, 1 case was HR-HPV positive with a SLE staining pattern (Fig. 3, d), which was consistent with the original CIN I diagnosis; the other case was negative, and the diagnosis was finally revised to immature squamous epithelium. Among the 5 CIN2 samples, 2 were HR-HPV positive. Among them, 1 case was consistent with the RNAscope staining characteristics of CIN2 in the control group and agreed with the original CIN2 diagnosis; however, another case showed full-thickness nuclei/cytoplasmic scattered dotted expression (FTE staining pattern), which was consistent with the staining characteristics of CIN3 in the control group. Therefore, the diagnosis of this case was revised from CIN2 to CIN3 (Fig. 3, h). The remaining 3 cases were negative, and the original diagnosis was revised to inflammatory changes. Additionally, the last case diagnosed as CIN3 was positive and showed a FTE staining pattern (Fig. 3, l), which is consistent with a CIN3 diagnosis.

#### Test-C group

**Fig. 4:**
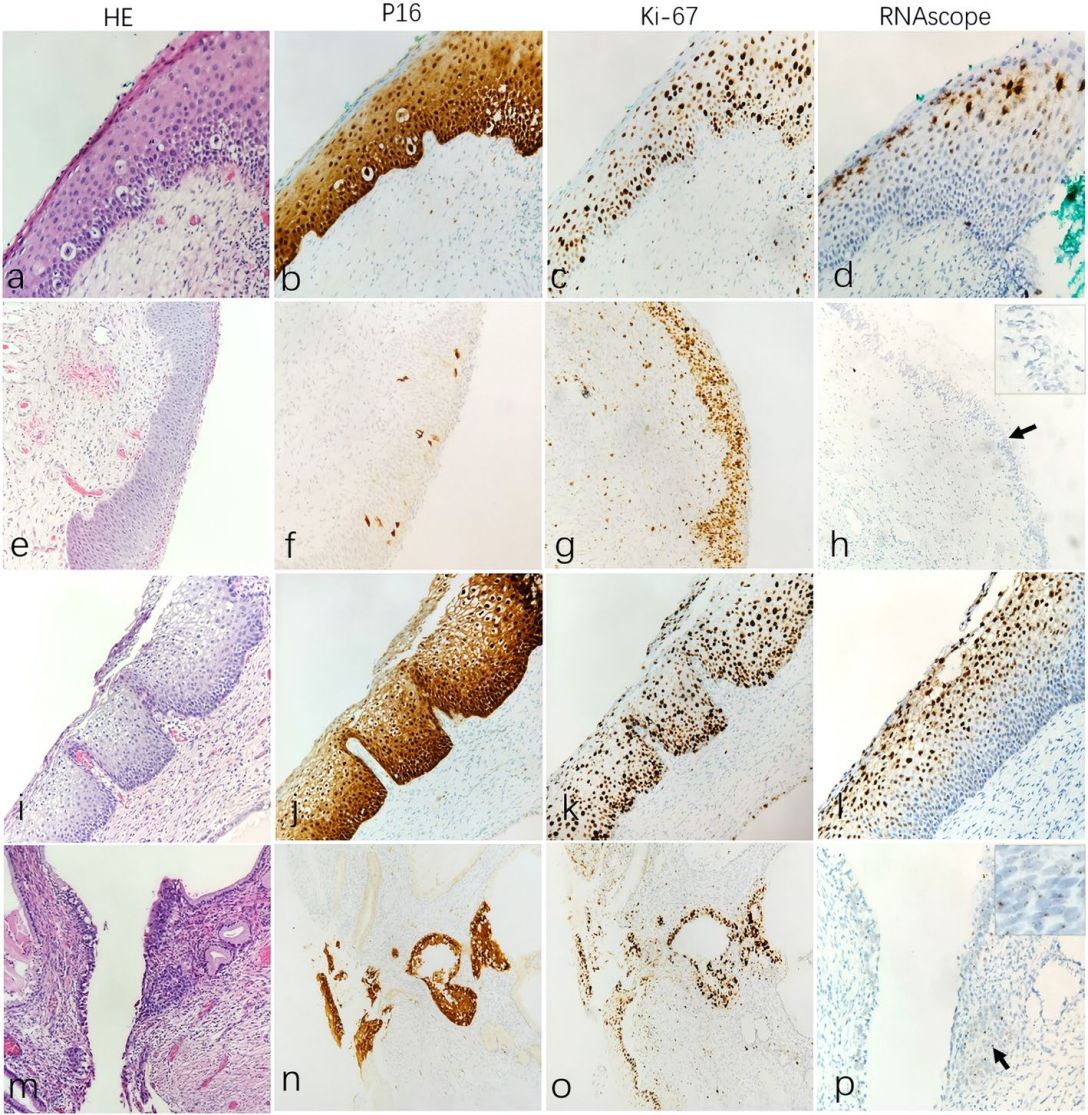
The H&E morphology, P16/Ki67 phenotype and RNAscope expression patterns in Test-C group. Row1 (**a**-**d**): HPV-DNA negative of CIN1 case but positive for both P16 and Ki67, RNAscope show diffuse clustered expression pattern in nuclei/cytoplasmic, and the staining areas is mainly located on the surface epithelium (**d**). Row2 (**e**–**h**): A case originally diagnosed as CIN1 had HPV-DNA + , P16- and Ki67 + phenotypes, but RNAscope is negative (**h**, arrow). Row3 (**i**-**l**): A case diagnosed as CIN2 had HPV-DNA-, P16 + and Ki67 + phenotypes, RNAscope show nucleus/cytoplasmic dot positivity in the basal and parabasal layer, and combined with diffuse cluster nuclei positivity on the surface epithelium (**l**). Row4 (**m**-**p**): A case diagnosed as CIN3 had HPV-DNA-, P16 + and Ki67 + phenotypes, RNAscope show dotted full-thickness nucleus/cytoplasmic scattered staining, combined with scattered punctate in CIN3-involving endocervical gland (**p**, arrow)

According to the results of HPV DNA detection, the inconsistent cases of HPV and P16/Ki-67 staining were included in the Test-C group, including the cases with P16 + , Ki-67 + , HPV- or P16-, Ki-67-, and HPV + staining. There were 15 cases. In this group, 3 cases were positive for HPV-DNA, including 1 case of CIN1 positive for 12 other HR-HPV types and 2 cases of CIN2 positive for 16 types and 12 other HR-HPV types. H&E morphology, P16 and Ki67 IHC and RNAscope characteristics are shown in Fig. 4. In 7 cases of original CIN1, 1 case was HR-HPV + and 1 case was LR-HPV + , and the staining pattern of LR-HPV + was SLE, agreeing with the original diagnosis; another HR-HPV + case had a focal staining pattern of FTE, and the diagnosis was corrected as CIN1 accompanied by focal CIN3 after the pathologist reviewed the H&E section again. In addition, the other 5 cases (including 1 case that was HPV-DNA positive for the other 12 HR-HPV types) were negative, and were revised to inflammatory or reparative changes (Fig. 4, h). There were 5 cases of CIN2 and only 1 case that was HPV-DNA- but HR-HPV + and showed the staining characteristics of CIN2 (Fig. 4, l). The remaining 4 cases (including 2 cases of HPV-DNA positivity for 16 and 12 other HR-HPV types) were all negative and subsequently revised to inflammatory lesions or reparative changes. For CIN3, 3 cases were negative for HPV-DNA, but both were positive for HR-HPV RNAscope, and the expression patterns were FTE and BME (basal to mid-epithelium staining pattern) (Fig. 4, p), which was consistent with the diagnosis of CIN3. The number and details of corrected diagnoses in each test group are summarized in Table 5.

**Table 5:** The number and details of corrected diagnosis cases in each test group

	Total	Corr	Detail
Test-A	13	7	
CIN1	4	2	CIN1 → Inflammatory or reparative changes
CIN2	6	5	CIN2 → Inflammatory or reparative changes
CIN3	3	0	
Test-B	8	5	
CIN1	2	1	CIN1 → Reparative changes
CIN2	5	4	3 cases CIN2 → Inflammatory or reparative changes
			1cases CIN2 → CIN3
CIN3	1	0	
Test-C	15	10	
CIN1	7	6	5 cases CIN1 → Inflammatory or reparative changes
			1 cases CIN1 → CIN3(Focal)
CIN2	5	4	CIN2 → Inflammatory or reparative changes
CIN3	3	0	

## Discussion

CIN diagnosis has always been puzzling for pathologists because of diagnostic subjectivity and uncertainty. For this reason, a number of auxiliary diagnostic techniques have been utilized. The most widely used are the HPV DNA test and P16/Ki67 immunohistochemistry. However, both of these tests have certain shortcomings. HPV DNA testing is conducted mainly through the collection of exfoliated cervical cells and uses the β-globin gene as an extraction and amplification control, based on real-time polymerase chain reaction (PCR) technology to detect whether the content of high-risk HPV DNA in cells exceeds the threshold [24–26]. The final results are only positive or negative, and it is difficult to identify HPV infection in a more intuitive way. Therefore, HPV DNA testing has insufficient specificity and the inability to be applied to FFPE samples [27–29]. Regarding IHC, P16 positivity and the high expression of Ki67 are generally suitable for the diagnosis of CIN lesions, but there are also some exceptions. For example, methylation of the p16 gene promoter can significantly reduce the expression of p16, cause it to lose its tumour suppressor activity and promote the development of CIN or UCC [30, 31]. In addition, Ki67 and P16 show transient increases and positive expression in the case of inflammatory injury and reparative hyperplasia of the cervix, which are easily misdiagnosed as CIN [20–22]. Furthermore, the interpretation of IHC is subjective, and there are no strict criteria to determine the final result. These conditions taken together (H&E slides, cytology, HPV-DNA and P16/Ki-67 IHC) can make it difficult to accurately assess CIN lesions. Recently, HPV RNAscope has been applied in the clinic as a reliable technique [32].

The pathogenic role of the HPV genotype in the development of CIN and cervical carcinoma has been well established [13, 33]. The oncogenic ability of HPV is mainly determined by viral E6/E7 proteins, and transcription and overexpression of specific E6/E7 genes eventually activate the downstream oncogenic signalling pathway, leading to the occurrence of CIN and UCC [34, 35]. The principle of HPV RNAscope is to use cascade signal amplification to detect the full-length or fragment of E6 and E7 transcripts [32, 36]. Therefore, the detection of E6/E7 mRNA transcripts offers the possibility of performing specific tests for CIN and UCC. However, the HPV RNA distribution and location in different CIN grades have not been described. Therefore, the present study attempted to determine the common expression of HPV RNA in the different grades of CIN. Then, we used this pattern to distinguish clinically difficult cases.

In this study, 85 lesions graded CIN 1, 2, or 3 after routine H&E review were investigated primarily by detecting HR and LR-HPV E6/E7 mRNA in combination with an HPV-DNA test and P16/Ki-67 IHC. There are three major findings of the present study.

First, there were different expression patterns of HPV E6/E7 mRNA in different CIN lesions. The main staining features of LSIL/CIN1 are diffuse clustered nuclear and cytoplasmic punctate expression in the superficial layer of epithelium (SLE + pattern). HSIL/CIN2 showed nuclear/cytoplasmic punctate or diffuse cluster nuclear staining in the mid-surface layer and scattered nuclear/cytoplasmic punctate staining in basal and parabasal cells (FTE/SLE + pattern). HSIL/CIN3 showed full-thickness nucleus/cytoplasmic scattered staining in the puncta (FTE + pattern). Interestingly, these different expression patterns maybe represented the different HPV infection phases in corresponding intraepithelial lesions. A large number of studies have shown that HPV infection is mainly divided into three stages: incubation, proliferation and transformation [37, 38]. Initially, HPV infects basal epithelial layer. The HPV virus in the incubation phase is generally difficult to detect because of its too low copy number in basal cells. In the proliferation phase (LSIL stage), the viral DNA replication does not depend on host cells and produces a large amount of replication product, so the mRNA particles become more abundant and accumulate in the superficial epithelium (SLE staining pattern). After a persistent infection of HPV, the process enters the transformation phase (HSIL stage); HPV DNA is integrated into the host genome and then replicates and divides with the host epithelial cells, eventually resulting in characteristic full-thickness mRNA expression (FTE staining pattern). As CIN2 is a transition from CIN1 to CIN3, both SLE and FTE staining patterns can be seen in CIN2.

The results show that the observation index of HPV RNAscope should focus on the location of mRNA particles expressed in tumour cells (including nucleus/cytoplasm and epithelium location), rather than the amount of cellular expression. The data show concordance between CIN grade and HPV infection stage, suggesting that HPV RNAscope may be a valuable auxiliary diagnostic test for CIN grading.

Second, the HPV RNAscope assay is a highly sensitive and specific method to directly confirm the stage of HPV infection in lesions by microscopy. Compared to the HPV DNA test, which uses PCR technology to confirm whether the HPV DNA content is higher than the threshold, the HPV RNAscope assay can screen more cases of HPV infection in a more intuitive and simple way.

In the data analysis of the Test-C group, we noticed that there were 3 cases of HSILs/CIN3 and 3 cases of HSILs/CIN2 having typical histological morphology, and P16/Ki67 was also in line with expectations (P16 diffuse strong or patchy-like positivity, Ki67 high expression), but HPV DNA was not detected in cervical cytology. However, we found scattered positive expression in tumour cell nuclei through HPV RNAscope in 4/5 cases (3 CIN3 and 1 CIN2). We speculated that HPV-DNA was negative in these 4 cases because the content did not reach the threshold during the testing process. However, under the microscope, we can see that even though the expression level is not high, HPV virus DNA has been clearly integrated into the host nucleus. This phenomenon demonstrates that HPV RNAscope has obvious advantages over the HPV-DNA test. In addition, HPV RNAscope also detected more high-risk or low-risk HPV phenotypes than HPV DNA tests. Therefore, we advocate that such cases need further testing with HPV RNAscope to determine HPV status and to assist our final diagnosis.

Another point worth noting is that among the 24 cases with HPV-DNA positivity in the Test group, only 10 cases were positive by HPV RNAscope analysis, and the positive rate was 41.7%. We speculate that the results are mainly related to transient HPV infection because the virus is then cleared by the body’s own immune system [39–41]. The virus is not involved in the cervical surface epithelium, so we cannot observe the presence of HPV virus particles in HPV RNAscope.

Third, the HPV RNAscope assay can effectively discriminate between true CIN and normal or CIN mimic lesions, such as immature squamous metaplasia, atrophy, and inflammatory/reactive changes. In this study, we corrected 20 cases originally diagnosed as CIN lesions (8 cases were CIN1 and 12 cases were CIN2) as inflammatory or repair/reactive changes after reviewing the HPV RNAscope results. Additionally, 1 case was changed from CIN1 to CIN3, and 1 case was changed from CIN2 to CIN3. This knowledge can reduce the cases of both non-CIN lesions being mistaken for CIN and CIN not being recognized, thus avoiding the overtreatment and undertreatment of CIN lesions, respectively. In addition, the HPV RNAscope staining pattern may be the first diagnostic tool supporting the histological basis of CIN2.

Inevitably, this study has some limitations. The main one was the relatively small sample size. In addition, we were unable to evaluate the risk of lesion progression associated with HPV E6/E7 mRNA because this was a cross-sectional study. It is necessary to conduct further prospective analysis and in-depth studies with a larger sample size in the future and strive to provide a more objective and convincing method for auxiliary cervical lesion classification.

## Conclusions

In conclusion, this study supports the viewpoint that HPV RNAscope can be used to assist in the grading of CINs. The positive expression pattern of HPV E6/E7 mRNA may reflect the stage of HPV infection and the developmental direction of CINs, providing the possibility for patients to achieve more precise treatment. In addition, newly trained or relatively inexperienced pathologists can rely on HPV RNAscope for the differential diagnosis of difficult CIN-related lesions, such as repair or immature squamous metaplasia. HPV RNAscope has great potential value for the pathological diagnosis of CINs and deserves further study. However, it is worth noting by pathologists that even though we have discussed the many advantages of HPV RNAscope above, morphology is the most important component of CIN lesions, and RNAscope is also carried out on the basis of morphology. Treating RNAscope as an auxiliary tool rather than a necessity can effectively prevent the occurrence of overdiagnosis/underdiagnosis of CINs.

## Supplementary Information

**Additional file 1.**

## Data Availability

The datasets used and/or analysed during the current study are available from the corresponding author on reasonable request.

## Abbreviations

CIN: Cervical intraepithelial neoplasia
HPV: Human papilloma virus
IHC: Immunohistochemistry
UCC: Uterine cervical carcinoma
LEEP: Loop electrosurgical excision procedure
SIL: Squamous intraepithelial lesions
WHO: World Health Organization
TCT: Thinprep cytologic test Squamous
FFPE: Formalin-fixed paraffin-embedded
H&E: Haematoxylin-Eosin
LAST: Lower Anogenital Terminology
PCR: Polymerase chain reaction
SLE: Superficial layer epithelium
FTE: Full thickness epithelium
